# Supplementary material for: Brain functional connectivity and anatomical features as predictors of cognitive behavioral therapy outcome for anxiety in youths
Source: Psychol Med. 2025 Mar 24;55:e91. doi: 10.1017/S0033291724003131 (PMC12080668; doi:10.1017/S0033291724003131)
Supplement: Zugman et al. supplementary material [file S0033291724003131sup001.docx]

Brain Functional Connectivity and Anatomical Features as Predictors of Cognitive Behavioral Therapy Outcome for Anxiety in Youths

**Supplementary Material**

Andre Zugman^1*^, Grace V. Ringlein^1^, Emily S. Finn^2^, Krystal M. Lewis^1^, Erin Berman^1^,
Wendy K. Silverman^3^, Eli R. Lebowitz^3^, Daniel S. Pine^1^ and Anderson M. Winkler^4^.

1. Emotion and Development Branch, National Institute of Mental Health, National Institutes of Health, 9000 Rockville Pike, Bethesda, MD, 20892, USA.

2. Psychological and Brain Sciences, Dartmouth College, 3 Maynard St, Hanover, NH, 03755, USA.

3. Child Study Center, Yale University, 230 South Frontage Rd., New Haven, CT 06520, USA.

4. Division of Human Genetics, School of Medicine, University of Texas Rio Grande Valley, 1 West University Blvd, Brownsville, TX 78520, USA.

*Corresponding author: Emotion and Development Branch, National Institute of Mental Health, National Institutes of Health, 9000 Rockville Pike, Bethesda, MD, 20892, USA. Telephone: +1 (301) 480-8395, email: andre.zugman@nih.gov

# Supplementary Methods

## Image acquisition

**Dataset A:** Scans were performed on two identical General Electric Signa Discovery MR750 3T scanners using a 32-channel head coil. Resting-state fMRI was acquired for 10 minutes (304 volumes) with a multi-echo planar sequence (TR: 2000 ms, TE: 14/28/42 ms, flip angle: 77°, FoV: 240x240 mm, matrix size: 64×64, 34 axial interleaved slices, bandwidth: 7812.5 Hz/Pixel, voxel size: 3.75×3.75×3.80 mm). Participants were instructed to keep their eyes open and focus on a white fixation cross in the middle of a black screen. A T1-weighted MPRAGE sequence was acquired in the same scanning session (TR: 7.66 ms, TE: 3.476 ms; TI: 900 ms^^[[1]](#footnote-0)^^, FoV: 256×256 mm, flip angle: 7°, voxel size: 1×1×1 mm). Patients were allowed to watch a movie of their choice during the non-functional part of the scanning session. Dataset A was used for fingerprinting, and for CPM and APM with cross-validation.

**Dataset B:** For the replication analysis, a dataset collected in one of same scanners was used, although these images used a different echo-planar sequence for the fMRI (TR: 2000 ms, TE: 30 ms (single echo), flip angle: 90°, FoV: 192x192 mm, matrix size: 64×64, 36 axial interleaved slices, bandwidth: 7812.5 Hz/Pixel, voxel size: 3.0×3.0×4.0 mm), that was also shorter (6 minutes, 180 volumes), as well as for the corresponding T1-weighted MPRAGE (TR: 7.82 ms, TE: 3.02 ms, TI: 725 ms, FoV: 256 × 256 mm, flip angle: 6°, voxel size: 0.86×0.86×1.20 mm). Patients were, likewise, allowed to watch a movie of their choice acquisition of the non-functional scans. Dataset B was used for external validation of CPM and APM.

## Image processing

For both datasets, images were preprocessed identically using fMRIPrep (v. 21.0.0) [(Esteban et al. 2019)](https://www.zotero.org/google-docs/?jBUDpj). Additional QC metrics were obtained with MRIQC (v. 0.16.1) [(Esteban et al. 2017)](https://www.zotero.org/google-docs/?x4BvEk). All relevant outputs were visually inspected. Subjects were excluded for excessive motion if they had a frame displacement mean of greater than 0.25 mm or more than 50% of frames with frame displacement greater than 0.25 mm, and appeared to be low quality based on visual inspection of the fMRIPrep outputs. Of the 66 patients in Dataset A available for the CPM and APM analyses, n = 12 were excluded based on these criteria, leaving n = 54 subjects for the primary analysis. Of the 87 (patients = 57) subjects available for the fingerprinting analyses, n = 21 (patients = 15) were excluded based on these criteria, leaving n = 66 (patients = 42). Of the 31 patients in Dataset B available for CPM and APM, 16 were excluded based on these criteria, leaving n = 15 patients.

Details of motion and demographics in each sample are in **Tables 1 and 2**, and in **Supplementary Tables 1 and 2**; demographics for the excluded participants can be found in **Supplementary Tables 3, 4, and 5**. The excluded group for the fingerprinting analysis were significantly lower in baseline age (p = 0.02) and follow-up age (p = 0.03) than the included groups but were not significantly different in age of scan in the in Dataset A of the CPM analysis (Dataset A: p = 0.32, Dataset B: p < 0.01). Removal of motion artifacts was performed by regressing out ICA-AROMA noise components [(Pruim et al. 2015)](https://www.zotero.org/google-docs/?lUamgz), global signal, white matter signal, and CSF signal from the images aligned to MNI space. We use global signal regression (GSR) given that it has been shown to increase the correlation between resting state functional connectivity and behavioral measures [(Li et al. 2019)](https://www.zotero.org/google-docs/?NRxfZc). Results without GSR are shown in the Supplementary Material.

## Functional connectivity

The processed images were parcellated, in surface space, according to the Schaefer atlas with 200 surface-based ROIs [(Schaefer et al. 2018)](https://www.zotero.org/google-docs/?67ydCI) (100 per hemisphere), grouped into 17 networks [(Yeo et al. 2011)](https://www.zotero.org/google-docs/?M9WVsU) and additional 16 FreeSurfer [(Fischl 2012)](https://www.zotero.org/google-docs/?IOWVMC) ROIs for subcortical structures (8 for each hemisphere). A correlation matrix representing the rsFC between every pair of regions was computed for each subject and each session using Pearson’s correlation ($r$), and converted to a z-score using Fisher’s transformation ($z$ = arctanh($r$)), resulting in 23,220 unique edges that were used for fingerprinting and for CPM. Two variants of rsFC were considered: simple correlations, as well as partial correlations, whereby the timecourses of all other regions are regressed out from the timecourses of every pair of nodes whose correlation is being computed.

# Supplementary Results

## Processing choices

Across all measures, fingerprinting allowed identification of subjects based on their brain imaging profiles at rates that far exceeded chance. Nevertheless, the accuracy of fingerprinting, as well as that of CPM/APM, varied according to choices made during processing. Global signal regression (GSR) improved fingerprinting accuracy (53/66 vs 46/66 for baseline used to identify follow-up; 56/66 vs. 48/66 for follow-up used to identify baseline) as well as CPM accuracy (cross-validation: MAE difference = 0.0903, p = 0.0003; external validation: MAE difference = 0.1005, p = 0.0001). For these comparisons we used MAE as it uses the same units of the predicted quantities (PARS), thus serving as a better proxy of the size of the effect attributable to these differences. Using partial correlations instead of simple correlations reduced accuracy of fingerprinting (53/66 vs 33/66 for baseline used to identify follow-up; 56/66 vs. 32/66 for follow-up used to identify baseline), but improved accuracy of CPM (cross-validation: MAE difference = 0.2101, p = 0.0001; external validation: MAE difference = 0.1154, p = 0.0002). However, other processing choices also affected accuracy, and the generally better models used full (not partial) correlations.

Likewise, the accuracy of APM and anatomical fingerprinting varied depending on processing choices, although with a less consistent pattern than for CPM. Accuracy was perfect or near perfect for area, thickness, curvature, and sulcal depth, with at least 64/66 correct identifications for all these measurements, with or without smoothing, but not as close to perfect for gray/white matter contrast (59/66 with smoothing, although 64/66 without). Gray/white matter contrast, however, generally produced the best APM results compared, for example, with thickness (cross-validation: MAE difference = 6.1119, p = 0.0001; external validation: MAE difference = 0.2528, p = 0.0001) or surface area (cross-validation: MAE difference = 1.2642, p = 0.0100; external validation: MAE difference = 0.1274, p = 0.0001). While smoothing reduced the accuracy of fingerprinting only for gray/white matter contrast without affecting the accuracy for other anatomical measures, it did, counterintuitively, reduce the accuracy of APM for most models and measurements considered. For gray/white matter contrast, smoothing in some cases led to no vertices being detected in the first stage of the predictive modeling.

For both CPM and APM, using (a) a regression model that did not include age, sex, and scanner (where applicable) as nuisance variables, (b) a model in which these were regressed out from data and design, or (c) a model in which these were used as predictors, led to sometimes improved or reduced MAE. Likewise, the choice of edges in the first stage of CPM as (a) positively correlated only, (b) negatively correlated only, (c) both, or (d) the edges with highest differential power from fingerprinting, also led to inconsistent improvements or reductions in the MAE. Weighting the edges by their p-value in logarithmic scale (a rough measure of effect size given the fixed sample sizes) also sometimes improved, sometimes reduced accuracy. The Supplementary Material to this paper includes a large spreadsheet file containing accuracy results (MAE, r, and R2) for CPM and APM, in both cases using cross-validation within Dataset A and external validation using Dataset B. In the spreadsheet, the columns represent:

- *filename:* A unique identifier for the text file containing the results shown in the corresponding row (not relevant for reading these results).
- *model:* Indicates whether the model included variables such as age, sex, and baseline PARS were used as predictors (predictors), as nuisance variables (residualized), or if omitted from the model (nonuisance).
- *meas (APM only):* Indicates what morphological variable was used for prediction: area (area), curvature (curv), sulcal depth (sulc), cortical thickness (thickness), or gray/white contrast (w-g.pct.mgh).
- *fwhm (APM only):* Indicates the amount of smoothing applied: no smoothing (0) or smoothing with a Gaussian kernel of full width at half maximum of 15 mm (15).
- *denoise (CPM only):* Indicates whether denoising used AROMA components and white matter and CSF signal (AROMA) or further included global signal regression (AROMA-GSR).
- *netmat (CPM only):* Indicates whether the connectivity matrix used for this model used partial correlations (partial) or not (full).
- *vertices (APM) or edges (CPM):* Indicates whether edges/vertices selected in the first regression of the predictive modeling were those with a positive (pos) correlation with PARS, negative (neg), or both (both), or if the edges/vertices selected were those with highest differential power from fingerprinting (finger).
- *weighted:* Indicates whether a simple sum of values from edges/vertices selected in the first stage of the predictive model was used (FALSE) or whether a weighted sum based on the negative logarithm of p-values was used (TRUE), thus giving stronger weight to more significant edges/vertices.
- *pars:* Indicates what PARS was tentatively predicted by the model: PARS at the start of treatment (i.e., week 0, parsTotalStart), PARS at the end of treatment (i.e., week 8 or 12 depending on the dataset, parsTotalEnd), the difference between PARS at start and end (parsDelta), or PARS at the end of treatment after taking the PARS at start as nuisance (BaselineAsNuisance). For BaselineAsNuisance, there are no models configured as nonuisance.
- *train, test (external validation only):* Indicators of what datasets were used for training or for testing (i.e., datasets A or B). The spreadsheet also includes training and testing on the same dataset; such models are expected to have excellent performance, and were run only for sanity checking; results are not meant to be used otherwise.
- *Rsq, Rsq_lowerCI, Rsq_upperCI:* Cross-validation R2, along with lower and upper bounds for the corresponding 95% bootstrap confidence intervals.
- *MAE, MAE_lowerCI, MAE_upperCI:* Mean absolute error, along with lower and upper bounds for the corresponding 95% bootstrap confidence intervals.
- *Corr, Corr_lowerCI, Corr_upperCI:* Correlation between observed and predicted values, along with lower and upper bounds for the corresponding 95% bootstrap confidence intervals.
- *NumberBootStraps:* Number of bootstraps used to produce the confidence intervals.
- *NumberOfVertices (APM) or NumberOfEdges (CPM):* For external validation, this is the number of vertices or edges selected in the first stage of the predictive modeling. For cross-validation, this is the average across leave-one-out folds. If there are zero edges or vertices selected in the first stage, models configured as predictors and residualized are expected to yield the same results.

Colors in the spreadsheet are conditional on the values shown, and convey no information other than already represented by the respective numbers.

# Supplementary Tables and Figures

## Supplementary Table 1: Detailed descriptive statistics for Datasets A and B, as used for the CPM and APM analyses.

|  | Dataset A (n = 54) | | Dataset B (n = 15) | |  |  |
| --- | --- | --- | --- | --- | --- | --- |
|  | Mean or n | SD or % | Mean or n | SD or % | Test statistic* | p-value |
| Age | 13.06 | 2.70 | 13.19 | 3.37 | -0.17 | 0.87 |
| Framewise displacement (mean) | 0.11 | 0.05 | 0.14 | 0.06 | -2.15 | 0.03 |
| Framewise displacement (%) | 9.79 | 8.88 | 15.40 | 13.34 | -1.93 | 0.06 |
| IQ (WASI) | 112.39 | 13.42 | 115.53 | 17.17 | -0.75 | 0.45 |
| PARS total at baseline | 14.63 | 2.84 | 16.73 | 2.89 | -2.53 | 0.01 |
| PARS total at follow-up | 8.93 | 4.44 | 13.73 | 3.37 | -3.88 | <0.01 |
| PARS difference | -5.70 | 4.39 | -3.00 | 3.98 | -2.15 | 0.04 |
| Sex: | | | | | 0.01 | 0.93 |
| Female | 33 | 61 % | 10 | 67 % |  |  |
| Male | 21 | 39 % | 5 | 33 % |  |  |
| Race (as reported): | | | | | 6.80 | 0.34 |
| American Indian or Alaskan Native | 1 | 2 % | 0 |  |  |  |
| Asian | 2 | 4 % | 1 | 7 % |  |  |
| Black or African American | 2 | 4 % | 2 | 13 % |  |  |
| Multiple Races | 8 | 15 % | 1 | 7 % |  |  |
| Unknown | 3 | 6 % | 1 | 7 % |  |  |
| White | 38 | 70 % | 9 | 60 % |  |  |
| Ethnicity: | | | | | 1.03 | 0.60 |
| Latino or Hispanic | 9 | 17 % | 2 | 13 % |  |  |
| Not Latino or Hispanic | 42 | 78 % | 13 | 87 % |  |  |
| Unknown | 3 | 6 % | 0 |  |  |  |
| Highest Educational level of the parents: | | | | | 3.34 | 0.50 |
| Graduate Level | 41 | 76 % | 10 | 67 % |  |  |
| Standar College | 8 | 15 % | 5 | 33 % |  |  |
| Partial College, High School or lower | 4 | 8 % | 0 |  |  |  |
| Income: | | | | | 9.39 | 0.05 |
| < $60,000 | 0 | 0% | 2 | 13% |  |  |
| $60,000.00 - $89,999.99 | 6 | 11 % | 4 | 27 % |  |  |
| $90,000.00 - $179,999.99 | 20 | 37 % | 4 | 27 % |  |  |
| > $180,000.00 | 24 | 44 % | 5 | 33 % |  |  |
| Diagnosis**: | | | | | 7.50 | 0.76 |
| Panic Disorder | 2.0 | 4 % | 0.0 | 0 % |  |  |
| Separation Anxiety | 17.0 | 31 % | 5.0 | 33 % |  |  |
| Social Phobia | 33.0 | 61 % | 10.0 | 67 % |  |  |
| Specific Phobia | 14.0 | 26 % | 3.0 | 20 % |  |  |
| Generalized Anxiety Disorder | 46.0 | 85 % | 11.0 | 73 % |  |  |

* Two-sample t-test or Chi-squared test when appropriate.

** Patients may have more than one diagnosis, thus the sum is higher than 100%.

## Supplementary Table 2: Comparison between included and excluded subjects in the CPM and APM analyses – Dataset A.

|  | Included (n = 54) | | Excluded (n = 12) | |  |  |
| --- | --- | --- | --- | --- | --- | --- |
|  | Mean or n | SD or % | Mean or n | SD or % | Test statistic* | p-value |
| Age | 13.06 | 2.70 | 12.17 | 3.26 | 0.99 | 0.32 |
| Framewise displacement (mean) | 0.11 | 0.05 | 0.70 | 0.83 | -5.32 | 0.00 |
| Framewise displacement (%) | 9.79 | 8.88 | 46.99 | 19.50 | -10.20 | 0.00 |
| IQ (WASI) | 112.39 | 13.42 | 118.33 | 11.53 | -1.42 | 0.16 |
| PARS total at baseline | 14.63 | 2.84 | 15.83 | 3.19 | -1.30 | 0.20 |
| PARS total at follow-up | 8.93 | 4.44 | 9.17 | 5.24 | -0.16 | 0.87 |
| PARS difference | -5.70 | 4.39 | -6.67 | 4.01 | 0.70 | 0.49 |
| Sex: | | | | | 0.15 | 0.70 |
| Female | 33 | 61 % | 6 | 50 % |  |  |
| Male | 21 | 39 % | 6 | 50 % |  |  |
| Race (as reported): | | | | | 2.42 | 0.79 |
| American Indian or Alaskan Native | 1 | 2 % | 0 |  |  |  |
| Asian | 2 | 4 % | 0 |  |  |  |
| Black or African American | 2 | 4 % | 0 |  |  |  |
| Multiple Races | 8 | 15 % | 3 | 25 % |  |  |
| Unknown | 3 | 6 % | 0 |  |  |  |
| White | 38 | 70 % | 9 | 75 % |  |  |
| Ethnicity: |  |  |  |  | 1.05 | 0.59 |
| Latino or Hispanic | 9 | 17 % | 3 | 25 % |  |  |
| Not Latino or Hispanic | 42 | 78 % | 9 | 75 % |  |  |
| Unknown | 3 | 6 % | 0 |  |  |  |
| Highest educational level of the parents: | | | | | 0.47 | 0.79 |
| Graduate Level | 41 | 76 % | 9 | 75 % |  |  |
| Standar College | 8 | 15 % | 2 | 17 % |  |  |
| Partial College, High School or lower | 2 | 4 % | 0 | 0 % |  |  |
| Income: | | | | | 1.40 | 0.50 |
| < $60,000 | 0 | 0 % | 0 | 0 % |  |  |
| $60,000.00 - $89,999.99 | 6 | 11 % | 1 | 8 % |  |  |
| $90,000.00 - $179,999.99 | 20 | 37 % | 6 | 50 % |  |  |
| > $180,000.00 | 24 | 44 % | 3 | 25 % |  |  |
| Diagnosis**: | | | | | 15.21 | 0.23 |
| Panic Disorder | 2.0 | 4 % | 1.0 | 8 % |  |  |
| Separation Anxiety | 17.0 | 31 % | 4.0 | 33 % |  |  |
| Social Phobia | 33.0 | 61 % | 7.0 | 58 % |  |  |
| Specific Phobia | 14.0 | 26 % | 5.0 | 42 % |  |  |
| Generalized Anxiety Disorder | 46.0 | 85 % | 10.0 | 83 % |  |  |

* Two-sample t-test or Chi-squared test when appropriate.

** Patients may have more than one diagnosis, thus the sum is higher than 100%.

## Supplementary Table 3: Comparison between included and excluded subjects in the CPM and APM analyses – Dataset B.

|  | Included (n = 15) | | Excluded (n = 16) | |  |  |
| --- | --- | --- | --- | --- | --- | --- |
|  | Mean or n | SD or % | Mean or n | SD or % | Test statistic* | p-value |
| Age | 13.19 | 3.37 | 10.33 | 1.24 | 3.18 | 0.00 |
| Framewise displacement (mean) | 0.14 | 0.06 | 0.77 | 0.58 | -4.15 | 0.00 |
| Framewise displacement (%) | 15.40 | 13.34 | 54.24 | 16.55 | -7.16 | 0.00 |
| IQ (WASI) | 115.53 | 17.17 | 109.00 | 18.04 | 1.02 | 0.32 |
| PARS total at baseline | 16.73 | 2.89 | 16.44 | 3.05 | 0.28 | 0.78 |
| PARS total at follow-up | 13.73 | 3.37 | 12.69 | 3.46 | 0.85 | 0.40 |
| PARS difference | -3.00 | 3.98 | -3.75 | 3.53 | 0.56 | 0.58 |
| Sex: | | | | | 0.02 | 0.89 |
| Female | 10 | 67 % | 10 | 62 % |  |  |
| Male | 5 | 33 % | 6 | 38 % |  |  |
| Race (as reported): | | | | | 2.50 | 0.78 |
| American Indian or Alaskan Native | 1 | 7 % | 0 |  |  |  |
| Asian | 2 | 13 % | 2 | 12 % |  |  |
| Black or African American | 1 | 7 % | 2 | 12 % |  |  |
| Multiple Races | 1 | 7 % | 0 |  |  |  |
| Unknown | 1 | 7 % | 1 | 6 % |  |  |
| White | 9 | 60 % | 11 | 69 % |  |  |
| Ethnicity: |  |  |  |  | 4.01 | 0.13 |
| Latino or Hispanic | 2 | 13 % | 0 |  |  |  |
| Not Latino or Hispanic | 13 | 87 % | 14 | 88 % |  |  |
| Highest educational level of the parents: | | | | | 0.00 | 1.00 |
| Graduate Level | 10 | 67 % | 11 | 69 % |  |  |
| Standar College | 5 | 33 % | 4 | 25 % |  |  |
| Partial College, High School or lower | 0 | 0 % | 0 | 0 % |  |  |
| Income: | | | | | 5.90 | 0.12 |
| < $60,000 | 2 | 13 % | 2 | 12 % |  |  |
| $60,000.00 - $89,999.99 | 4 | 27 % | 0 | 0 % |  |  |
| $90,000.00 - $179,999.99 | 4 | 27 % | 9 | 56 % |  |  |
| > $180,000.00 | 5 | 33 % | 5 | 31 % |  |  |
| Diagnosis**: | | | | | 8.04 | 0.53 |
| Panic Disorder | 0.0 | 0 % | 0.0 | 0 % |  |  |
| Separation Anxiety | 5.0 | 33 % | 9.0 | 56 % |  |  |
| Social Phobia | 10.0 | 67 % | 8.0 | 50 % |  |  |
| Specific Phobia | 3.0 | 20 % | 4.0 | 25 % |  |  |

* Two-sample t-test or Chi-squared test when appropriate.

** Patients may have more than one diagnosis, thus the sum is higher than 100%.

## Supplementary Table 4 : Comparison between included and excluded subjects in the fingerprinting analyses.

|  | Included (n = 66) | | Excluded (n = 21) | |  |  |
| --- | --- | --- | --- | --- | --- | --- |
|  | Mean or n | SD or % | Mean or n | SD or % | Test statistic* | p-value |
| Age at baseline | 13.86 | 2.59 | 12.29 | 2.93 | 2.30 | 0.02 |
| Age at follow-up | 14.14 | 2.56 | 12.68 | 2.97 | 2.14 | 0.03 |
| Time between scans (days) | 122.78 | 40.37 | 142.76 | 55.55 | -1.79 | 0.08 |
| IQ | 111.52 | 12.83 | 116.37 | 11.49 | -1.49 | 0.14 |
| Sex: | | | | | 0.21 | 0.64 |
| Female | 39 | 59 % | 10 | 48 % |  |  |
| Male | 27 | 41 % | 10 | 48 % |  |  |
| Race (as reported): | | | | | 2.25 | 0.81 |
| American Indian or Alaskan Native | 1 | 2 % | 0 |  |  |  |
| Asian | 4 | 6 % | 1 | 5 % |  |  |
| Black or African American | 6 | 9 % | 1 | 5 % |  |  |
| Multiple Races | 13 | 20 % | 4 | 19 % |  |  |
| Unknown | 4 | 6 % | 0 |  |  |  |
| White | 38 | 58 % | 14 | 67 % |  |  |
| Ethnicity: | | | | | 0.71 | 0.70 |
| Latino or Hispanic | 8 | 12 % | 3 | 14 % |  |  |
| Not Latino or Hispanic | 56 | 85 % | 17 | 81 % |  |  |
| Unknown | 2 | 3 % | 0 |  |  |  |
| Highest educational level of the parents: | | | | | 1.87 | 0.39 |
| Graduate Level | 42 | 64 % | 13 | 62 % |  |  |
| Standar College | 12 | 18 % | 5 | 24 % |  |  |
| Partial College, High School or lower | 5 | 8 % | 0 | 0 % |  |  |
| Any psychiatric diagnosis: | | | | | 1.20 | 0.27 |
| Yes | 42 | 64 % | 16 | 76 % |  |  |
| No | 24 | 36 % | 4 | 19 % |  |  |
| Income: | | | | | 5.78 | 0.12 |
| < $60,000.00 | 5 | 8 % | 0 | 0 % |  |  |
| $60,000.00 - $89,999.99 | 5 | 8 % | 3 | 14 % |  |  |
| $90,000.00 - $179,999.99 | 21 | 32 % | 10 | 48 % |  |  |
| > $180,000.00 | 27 | 41 % | 4 | 19 % |  |  |

* Two-sample t-test or Chi-squared test when appropriate.

## Supplementary Table 5: Detailed descriptive statistics for the fingerprinting sample. In the fingerprinting analysis HV were included to maximize the sample with baseline and follow-up scans.

|  | Patients (n = 42) | | HV (n = 24) | |  |  |
| --- | --- | --- | --- | --- | --- | --- |
|  | Mean or n | SD or % | Mean or n | SD or % | Test statistic * | p-value |
| Time between scans (days) | 128.20 | 40.68 | 113.54 | 38.93 | 1.42 | 0.16 |
| Age at baseline | 13.67 | 2.67 | 14.19 | 2.47 | -0.78 | 0.44 |
| Age at follow-up | 13.93 | 2.63 | 14.50 | 2.45 | -0.86 | 0.39 |
| FD (mean) at baseline | 0.11 | 0.05 | 0.10 | 0.05 | 0.17 | 0.87 |
| FD (%) at baseline | 9.20 | 9.23 | 9.88 | 11.86 | -0.26 | 0.79 |
| FD (mean) at follow-up | 0.12 | 0.05 | 0.11 | 0.05 | 0.49 | 0.62 |
| FD (%) at follow-up | 10.21 | 9.51 | 10.24 | 10.07 | -0.01 | 0.99 |
| IQ (WASI) | 111.33 | 13.06 | 111.83 | 12.67 | -0.15 | 0.88 |
| Sex: | | | | | 0.77 | 0.38 |
| Female | 27 | 64 % | 12 | 50 % |  |  |
| Male | 15 | 36 % | 12 | 50 % |  |  |
| Race (as reported): | | | | | 7.76 | 0.17 |
| American Indian or Alaskan Native | 1 | 2 % | 0 |  |  |  |
| Asian | 2 | 5 % | 2 | 8 % |  |  |
| Black or African American | 1 | 2 % | 5 | 21 % |  |  |
| Multiple Races | 8 | 19 % | 5 | 21 % |  |  |
| Unknown | 3 | 7 % | 1 | 4 % |  |  |
| White | 27 | 64 % | 11 | 46 % |  |  |
| Ethnicity: | | | | | 1.80 | 0.41 |
| Latino or Hispanic | 6 | 14 % | 2 | 8 % |  |  |
| Not Latino or Hispanic | 34 | 81 % | 22 | 92 % |  |  |
| Unknown | 2 | 5 % | 0 |  |  |  |
| Highest educational level of the parents: | | | | | 6.95 | 0.03 |
| Graduate Level | 34 | 81 % | 8 | 33 % |  |  |
| Standar College | 6 | 14 % | 6 | 25 % |  |  |
| Partial College, High School or lower | 2 | 5 % | 3 | 12 % |  |  |
| Income: | | | | | 7.07 | 0.07 |
| < $60,000 | 1 | 2 % | 4 | 17 % |  |  |
| $60,000.00 - $89,999.99 | 4 | 10 % | 1 | 4 % |  |  |
| $90,000.00 - $179,999.99 | 13 | 31 % | 8 | 33 % |  |  |
| > $180,000.00 | 21 | 50 % | 6 | 25 % |  |  |
| Diagnosis**: | | | | |  |  |
| Panic Disorder | 2 % | 5 % |  |  |  |  |
| Separation Anxiety | 11 % | 26 % |  |  |  |  |
| Social Phobia | 27 % | 64 % |  |  |  |  |
| Specific Phobia | 13 % | 31 % |  |  |  |  |
| Generalized Anxiety Disorder | 36 % | 86 % |  |  |  |  |

* Two-sample t-test or Chi-squared test when appropriate.

** Patients may have more than one diagnosis, thus the sum is higher than 100%.

## Supplementary Figure 1: Pairplot graph of DP (differential power) by modality.


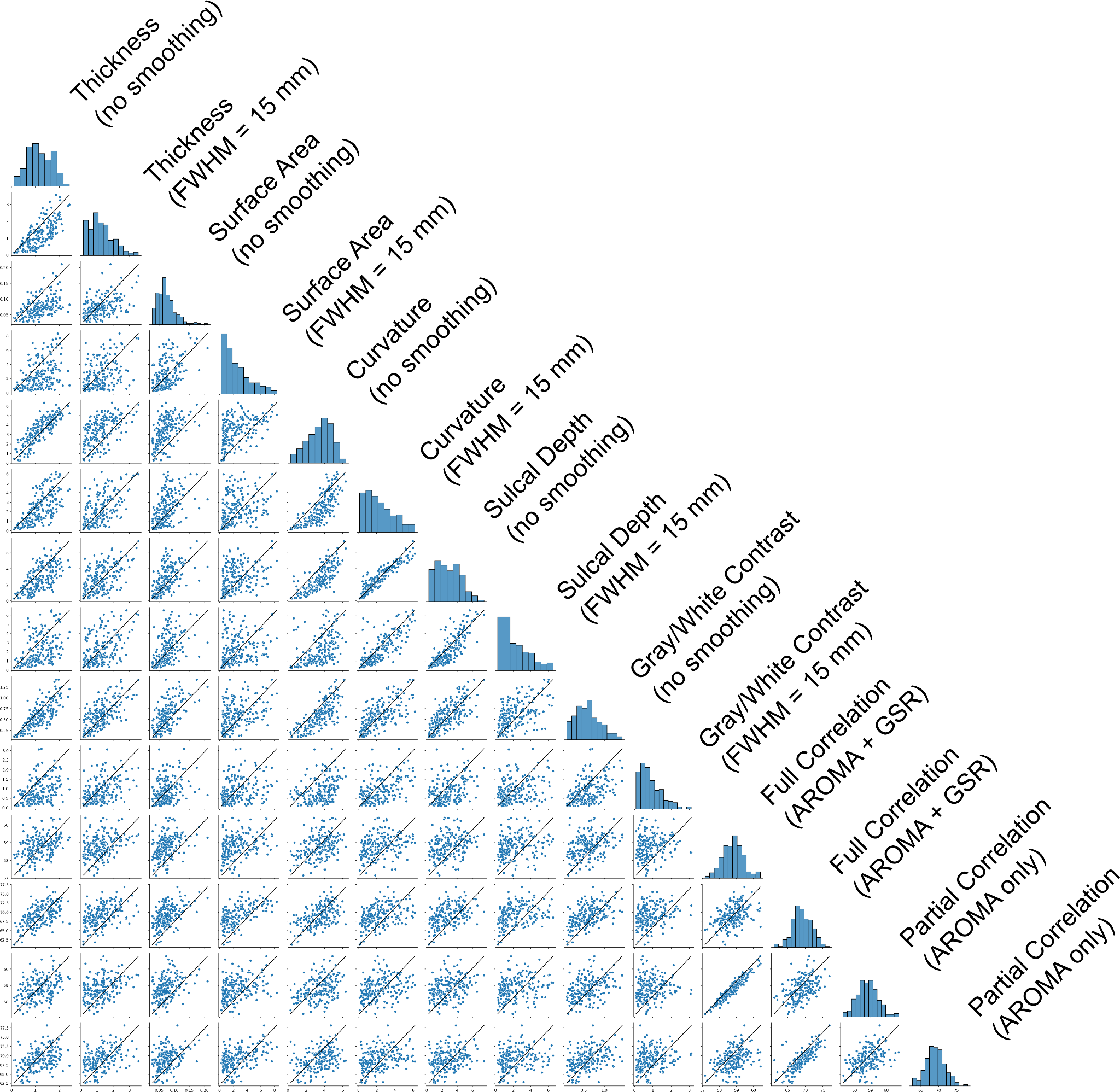


Pairplot graph of DP (differential power) by modality, including anatomical measurements (thickness, surface area, curvature, sulcal depth, and gray/white contrast) and functional measurements (connectivity assessed with full or partial correlations). For anatomical measurements, results for two levels of smoothing are shown; for functional, results are shown for denoising with or without global signal regression (GSR). Scatterplots represent the DP in 200 cortical ROIs in each modality. For vertex-wise data, the DP for all vertices within the ROI were averaged. FC is the average value of the average DP for all edges that represents connection to the ROI. The diagonal displays the histogram for the ROI DP in each modality.

## Supplementary Figure 2: Pairplot graph of DP (differential power) by modality with network hues.
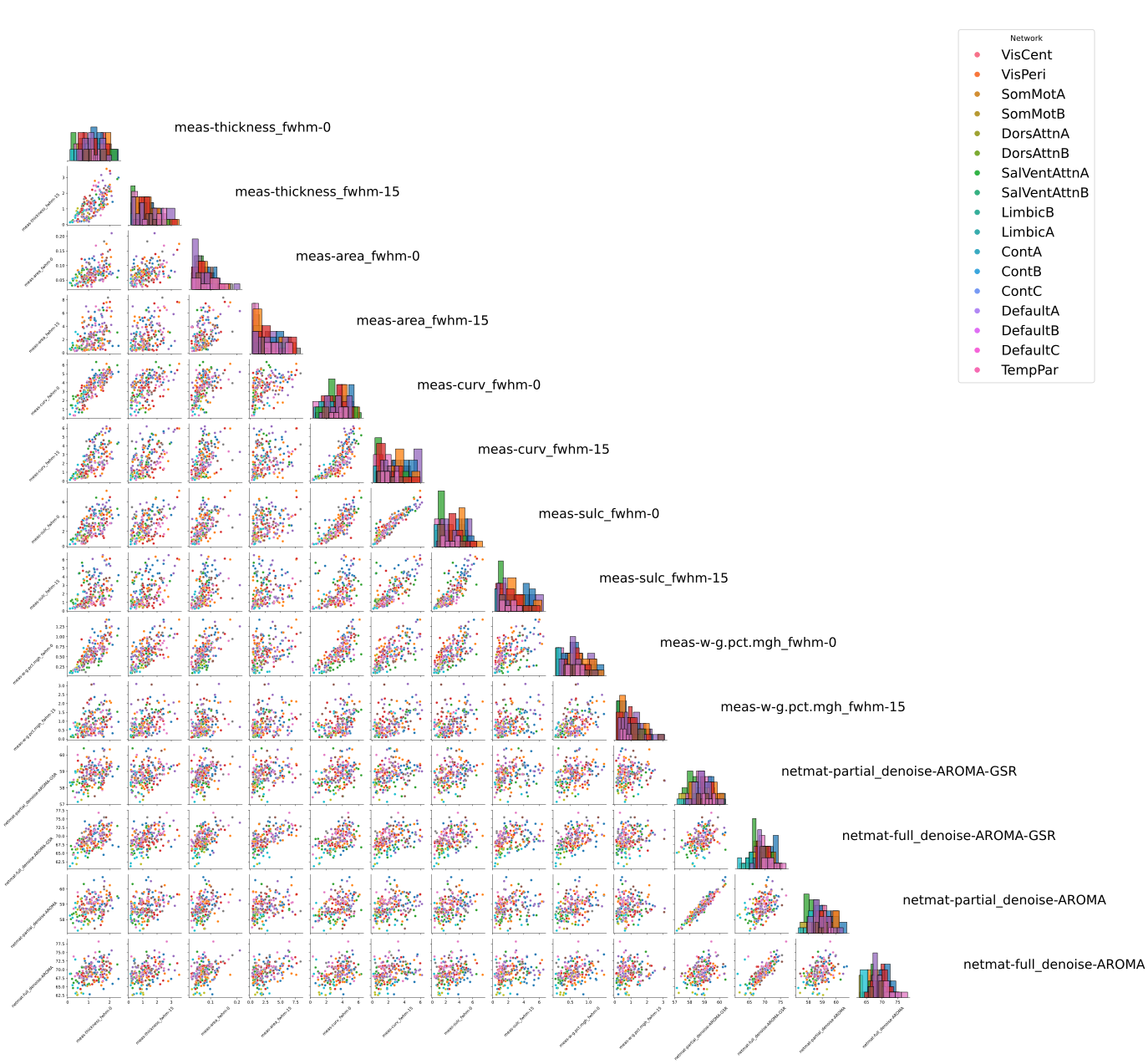


Pairplot of DP (differential power) by modality as in supplementary figure 1 showing different hues for each network in the Schaefer atlas. There is not a network that have a higher DP correlation between modalities.

##

## Supplementary Figure 3: Positive connections of figure 3 by network


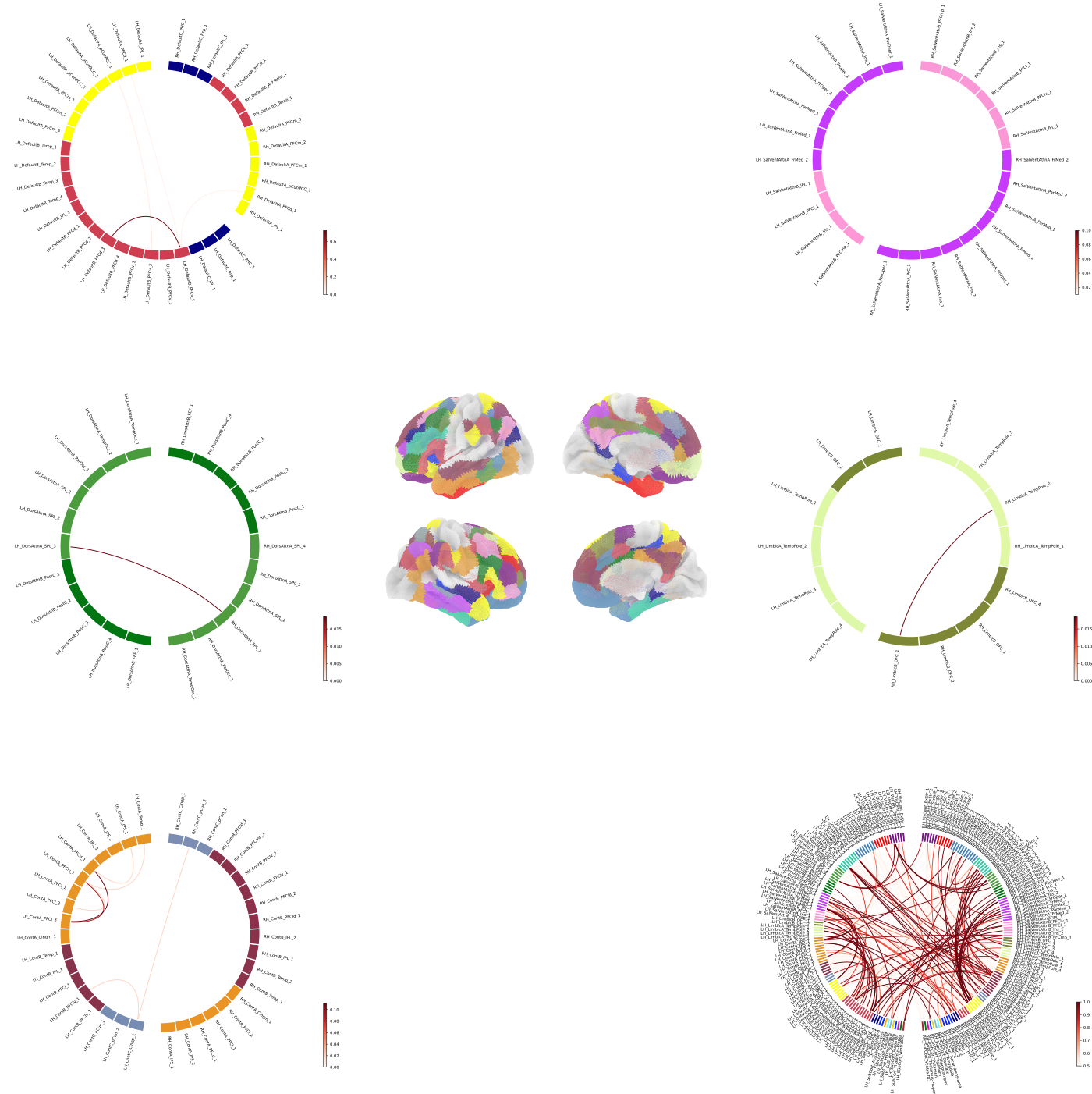


Positive edges as shown in Figure 3 of the main manuscript broken down by network. Within network connections were selected infrequently. In the case of Salience/Ventral Attention network, no within connection was selected in any of the CPM iterations. The bottom right plot shows between network connections were more frequently included for prediction. top left: default mode networks (A,B,C); top right: salience/ventral attention networks (A and B); middle right: dorsal attentional networks (A and B); middle left: limbic networks (A and B); bottom left: control networks (A,B,C); bottom right: between networks connections only.

1. 5 subjects in the final sample had TE = 3.42 ms and TI = 425 ms; the other parameters were the same. [↑](#footnote-ref-0)
